# Do rapid detection and isolation of colonized patients reduce MRSA spread in the intensive care unit?

**DOI:** 10.1186/1753-6561-5-S6-O88

**Published:** 2011-06-29

**Authors:** C Marshall, E McBryde, M Richards

**Affiliations:** 1 Victorian Infectious Diseases Service, Royal Melbourne Hospital, Melbourne, Australia; 2 Department of Medicine, University of Melbourne, Melbourne, Australia

## Introduction / objectives

Many studies have examined MRSA control measures, but few have been planned prospective single intervention studies in the endemic setting. We asked whether active surveillance & contact precautions (CP) reduced MRSA transmission. We collected & analysed data to avoid confounding (of MRSA acquisition with length of stay) & serial dependence due to colonisation pressure (CoPr) in accordance with ORION guidelines.

## Methods

This was an interrupted time series in a tertiary ICU. Standard precautions only were used for control patients regardless of MRSA status. In the intervention period, rapid PCR was used to detect MRSA colonised patients, who were then cared for in CP. We used a generalised estimating equation (GEE) with each patient-day being the unit of interest. The predictor variables were both time-dependent (antibiotic usage, hand hygiene & infection control precaution compliance & CoPr) & time-invariant covariates (co-morbidities, sex & age) & intra-individual risk correlation was adjusted for using the GEE framework.

## Results

The primary outcome measure was the effect of the intervention on MRSA acquisition, adjusting for CoPr & individual & ward covariates. The relative risk of MRSA acquisition was 0.40 (95%CI 0.24-0.65). Secondary measures include the effect of colonised patients in the ward (RR 4.38; 95% CI 1.00-19.21) & the phase-colonised patient interaction, with a reduced risk from colonised patients in the intervention phase to 0.41 (95% CI 0.14-1.18) compared with the control phase.

## Conclusion

We demonstrated a clinically significant effect of isolation on MRSA transmission. Our planned single intervention study is unique because of near-complete observations due to short intervals between swabs & full documentation of CoPr & time varying risk factors.

## Disclosure of interest

None declared.

